# A facility-based study of lipids, glucose levels and their correlates among pregnant women in public hospitals of northern Ethiopia

**DOI:** 10.1371/journal.pone.0279595

**Published:** 2023-06-06

**Authors:** Gebregziabher Berihu, Mengistu Mitiku, Selamawit Asfaw Beyene, Letekirstos Gebregziabher, Yohana Gebregiorgis, Mulu Eyasu, Molla Teferi, Tsegay Wellay, Alemtsehay Tewele, Mussie Tesfay Atsbeha

**Affiliations:** 1 Department of Health Systems, School of Public Health, College of Health Sciences, Mekelle University, Mekelle, Ethiopia; 2 Research and Community Engagement Coordination office, College of Health Sciences, Mekelle University, Mekelle, Ethiopia; 3 Department of Public Health Nutrition and Dietetics, School of Public Health, College of Health Sciences, Mekelle University, Mekelle, Ethiopia; 4 Department of Biostatistics, School of Public Health, College of Health Sciences, Mekelle University, Mekelle, Ethiopia; 5 Department of Environmental Health and Behavioural Sciences, School of Public Health, College of Health Sciences, Mekelle University, Mekelle, Ethiopia; 6 Department of Reproductive Health, School of Public Health, College of Health Sciences, Mekelle University, Mekelle, Ethiopia; 7 Department of Anaesthesia, School of Medicine, College of Health Sciences, Mekelle University, Mekelle, Ethiopia; Wachemo University, INDIA

## Abstract

**Background:**

Lipids and glucose concentrations in the blood rise during pregnancy period. Poor control of these analytes results in cardio metabolic dysfunction. Despite this, there are no documented studies which investigate lipids and glucose among pregnant women in Tigrai, northern Ethiopia.

**Objective:**

The objective of this study was to assess lipid and glucose levels and identify their correlates among pregnant women in Tigrai, northern Ethiopia.

**Method:**

We conducted a facility-based cross sectional study comprising of systematically selected 200 pregnant women from July to October 2021. Those who were severely ill were excluded from the study. We used a structured questionnaire to collect socio-demographic and clinical characteristics of pregnant women. Lipids such as triglycerides, low density lipoprotein, cholesterol and blood glucose were also measured using Cobas C311 chemistry machine from plasma samples. The data were analyzed using SPSS version 25. Logistic regression was performed and statistical significance was declared at p-value < 0.05.

**Result:**

Proportion of pregnant women with cholesterol, triglyceride, low density lipoprotein and blood glucose levels above the upper limit of the normal range used for clinical decision were 26.5%, 43%, 44.5% and 21%, respectively. Pregnant women’s income > = 10,000 ETB (AOR = 3.35; 95%CI: 1.46–7.66), age (AOR = 3.16; 95%CI: 1.03–9.68), gestational age 29–37 weeks (AOR = 8.02; 95%CI: 2.69–23.90) and having systolic blood pressure greater than 120 mmHg (AOR = 3.99; 95%CI: 1.64–9.75) demonstrated statistically significant association with raised levels of lipids.

**Conclusion and recommendation:**

Proportion of pregnant women with out of normal range values of lipids, particularly triglycerides and low density lipoprotein, is high. Gestational age is a strong predictor of increase in blood levels for both lipids. Provision of life style related health education and dietary intake to pregnant mothers matters. Moreover, monitoring lipid profile and glucose level during antenatal care period is essential.

## Introduction

Lipids and glucose, which are available at different concentrations in the body, are very essential ingredients for the growth and development of human being [1]. During physiologic pregnancy, progressive elevation of lipids and glucose takes place to meet the necessities for fetal growth and development [2, 3]. Such changes in glucose and lipid concentration occur to safeguard uninterrupted supply of nutrients to the developing fetus despite irregular maternal food intakes [4–6].

During gestational period, particularly the second and third trimesters, considerable metabolism activity of lipid and lipoprotein takes place and every serum lipid fraction such as triglycerides and glucose progressively increase. Sometimes, the plasma levels of those lipids increase to three fold of the baseline level [7, 8]. Moreover, a minor increase in total cholesterol, high and low density lipoprotein usually takes place [9, 10].

There are bad consequences associated with abnormal level of lipids and glucose. High level of total cholesterol, triglycerides and low-density lipoprotein are believed to be risk factors for the initiation and progression of atherosclerosis, eventually leading to cardiovascular diseases [11–13]. Besides, elevated physiological concentrations of maternal lipids in early or late pregnancy have revealed to result in adverse pregnancy outcomes. In the early periods of pregnancy, raised concentrations of maternal triglycerides are linked to preterm birth [14–16], pregnancy-induced hypertension [15, 17], gestational diabetes mellitus [18] and preeclampsia [15, 19–23]. In late periods of pregnancy, elevated concentrations of maternal lipids are associated with pregnancy induced hypertension [24] and preeclampsia [20, 22, 24]. Elevated blood glucose level is linked to slow and decreased foetal growth in mid-pregnancy and increased fetal growth rates from late pregnancy onwards, and an increased risk of delivering a large-for-gestational-age infant [25]. Moreover, understanding glucose level at the first antenatal care visit is shown to provide a clue in the prediction of gestational diabetes mellitus at later periods of pregnancy [26].

Despite all those consequences of increased level of lipids and glucose during pregnancy period and the availability of automated analysers in general and specialized hospitals, little attention is being given to measuring and monitoring of the most common and important lipids of pregnant women starting the early period of their antenatal care follow up in Tigrai, northern Ethiopia. To the best of our knowledge as healthcare providers, studies which evaluate lipids during pregnancy periods have never been done in Tigrai. Therefore, this study which used automated, internally and externally quality controlled chemistry analyzers, was proposed to assess the level and associated factors of lipids and blood glucose levels among pregnant women attending antenatal care services in two largest hospitals of Tigrai, Northern Ethiopia.

## Methods and materials

### Study design, study area and period

We conducted a facility-based cross-sectional study from July to October, 2021 in two government hospitals of Northern Ethiopia, namely: Ayder Comprehensive Specialized hospital and Mekelle general hospital. Ayder Comprehensive Specialized hospital is the biggest hospital in Northern Ethiopia and the second biggest in Ethiopia. The annual service delivery capacity of the hospital has exceeded 300,000 patients of which 5,000 were mothers who received childbirth services. Mekelle general hospital is the biggest of the sixteen general hospitals in Tigrai, Northern Ethiopia. The hospital currently serves patients referred from other general hospitals of Tigrai, in addition to its catchment area. The Ethiopian healthcare system is composed of three tier system. These are 1) Tertiary level healthcare which includes specialized hospital, 2) Secondary level healthcare which includes General hospitals and 3) Primary level healthcare which includes health centers only in urban areas and primary hospital, health center and health post in rural areas [27].

### Study population, sample size and sampling technique

The study subjects were pregnant women attending antenatal care clinic in the study hospitals. The required sample size was calculated using single population proportion formula and taking in to consideration the following assumptions: 95% confidence interval, 80% power, 5% significance level and 27% proportion of pregnant women with increased level of Triglycerides [28]. The calculated sample size was 302. However, due to the COVID-19 pandemic and the on-going war in Tigrai, we recruited two hundred pregnant women who came for antenatal care follow up services in the study hospitals. During the recruitment process, those who were severely ill and unable to communicate were excluded from our study. The number of pregnant women was allotted to the study hospitals based on proportional to size allocation and considering previous three months antenatal care clients flow. Hence, we learned that Ayder Comprehensive Specialized hospital served 450 pregnant women per month and Mekelle general hospital 300. Accordingly, the allotted sample size is 181 for Ayder and 121 for Mekelle hospitals. We applied systematic sampling method to recruit pregnant women.

### Study variables and operational definitions

Cholesterol, triglycerides, low density lipoprotein and glucose were the dependent variables. The independent variables were: socio-demographic factors like age, occupation and educational level; clinical variables like family history of diabetes, gravidity and Body Mass Index (BMI). Classification of the BMI was based on the recommendations of Center for Disease Control and Prevention (CDC) as follows: Underweight: BMI < 18.5 kg/m2; Normal weight: 18.5 ≤ BMI < 24.9 kg/m2; Overweight: 25 ≤ BMI < 29.9kg/m2; obese: BMI ≥ 30kg/m2 [29]. Lipid profile level refers to fasting blood cholesterol, low density lipoprotein, triglyceride; Upper limits refers to the following under fasting conditions: Cholesterol (>200 mg/dl), triglycerides (>150 mg/dl), low density lipoprotein (>100 mg/dl) and fasting blood glucose (>106 mg/dl).

### Data collection tool and procedure

Pregnant women’s socio-demographic and clinical variables were captured directly from the subjects using a structured questionnaire developed by making use of existing literatures [2, 6, 13, 18]. The laboratory investigation was carried out at the central laboratory of Ayder Comprehensive Specialized hospital. We used Cobas C311 Clinical Chemistry Analyzer to determine glucose and lipid concentrations.

### Data collection procedure

The research team trained data collectors and supervisors on how to give general information on the objective of the study, approach and treat study participants, and take written informed consent. From each study hospital, we recruited a physician and two midwife nurses as pregnant women assessors and data collectors, respectively. Besides, medical laboratory technologists were employed to collect blood samples and measure lipids and glucose concentration. Members of this project acted as supervisors of the data collection process. They were responsible for checking the daily activity, consistency and completeness of the filled questionnaire and giving appropriate support to data collectors. Incomplete and unclearly filled questionnaires were immediately given back to the physician, midwife nurse and laboratory technologists and got completed.

### Measurements

#### Specimen collection, plasma glucose and lipid concentration determination

Senior medical laboratory technologist collected blood samples at the central laboratories of both hospitals. Five millilitres of venous blood were drawn from each pregnant woman in to test tubes containing citrate anticoagulant for lipids and Ethylene Diamine Tetra Acetic acid (EDTA) for glucose determination after thoroughly cleaning their front arm using cotton soaked in 70% alcohol. Samples were centrifuged at 800 revolutions per minute for 6 minutes and stored at 2–8 degree centigrade before being assayed. Glucose and lipids levels were quantified using enzymatic colorimetric methods (an Automated Clinical Chemistry Analyser called ‘Cobas C311 was used).

#### Data quality management

Pre-test was done two weeks before the commencement of the actual study on 5% of the calculated sample at Wukro general hospital, located at least 45 kilometers away from one of the study hospitals and necessary corrections were made on the data collection tool based on the results of the pre-test. Members of the research team conducted on spot-checking of filled questionnaires and investigated results for errors or any incompleteness.

In the laboratory, quality of lipids and glucose concentration determinations were ensured through the collection of appropriate blood sample by trained medical laboratory technologists. These medical laboratory technologists were were made to have know-how on the adequacy and representativeness of samples taken from the pregnant women. Moreover, the validity and precision of the chemistry machine was checked using control samples obtained from Tigrai Health Research Institute, the only research institute in Tigrai, Northern Ethiopia. Moreover, control samples of the Cobas C311 chemistry analyser itself were used to check the accuracy and precision of the machine before lipid and glucose measurements of our samples were made.

### Data analysis

We entered the data to, cleaned and analyzed using SPSS version 25. Frequencies and percentages were developed for categorical variables. Association between the independent variables with lipids and glucose levels was checked using logistic regression at 5% significance level and 95% confidence interval. A *p*-value of less than 0.05 was considered statistically significant.

### Ethical consideration

The study was conducted in accordance with the Declaration of Helsinki. Study participants were informed about the purpose of the study, the procedure of venous blood sample collection and sample handling. Written informed consent was also obtained from voluntary pregnant women. Moreover, pregnant women’s data were kept with strict privacy. Ethical clearance with registration number of ERC 1493/2020 was obtained from Mekelle University, College of Health Science’s Institutional review board on October 4, 2020.

## Results

### Socio-demographic characteristics of pregnant women

In this study, two hundred pregnant women were enrolled. The response rate was 98.52% (three pregnant women refused to participate due to personal reasons). No pregnant women were excluded from our study (all systematically selected and voluntary pregnant women were included). Around half, 88 (44%) of the pregnant women were in the age range 26–30 years. One hundred seven (53.5%) pregnant women achieved at least seventh grade and utmost twelve grade. Moreover, two-third (71%) were house wives and 116 (58%) had an income level less than or equal to 5,000 Ethiopian birr. Majority of the pregnant women (81%) didn’t drink beer; around half (56%) drink ‘SIWA’, a traditional alcohol drink common in Tigrai, northern Ethiopia (Table 1).

**Table 1:** Socio-demographic characteristics of pregnant women in northern Ethiopian public hospitals, 2021 [n = 200].

Variable	Frequency	Percent
Age in years		
18–25	66	33
26–30	88	44
31–38	46	23
Total	200	100
Educational status		
1–6 grades	18	9
7–12 grades	107	53.5
>12 grades	75	37.5
Total	200	100
Occupation		
Government employee	40	20
House wife	142	71
Others*	18	9
Total	200	100
Income		
< = 5,000 ETB	116	58
5,000–9999 ETB	52	26
> = 10,000 ETB	32	16
Beer taking habit		
Yes	38	19
No	162	81
Total	200	100
‘SIWA’ taking habit		
Yes	112	56
No	88	44
Total	200	100
Physical activity		
Yes	26	13
No	174	87
Total	200	100

* = merchants, farmers, college students.

### Clinical data of pregnant women

One hundred sixteen (58%) pregnant women were with gestational age 17–28 weeks. Around two-third (66%) had a parity of one and greater. With regard to BMI, 106 (53%) were within the normal range. Majority of the pregnant women (90.5%) didn’t have maternal history of hypertension. Most of the pregnant women (83%) and 186 (93%) had a normal systolic and diastolic blood pressure, respectively (Table 2).

**Table 2:** Clinical data of pregnant women in northern Ethiopia public hospitals, 2021 [n = 200].

Variable	Frequency (N)	Percent (%)
Gestational age		
1–16 weeks	54	27
17–28 weeks	116	58
29–41 weeks	30	15
Total	200	100
Gravidity		
< = 1	46	23
2–3	120	60
> = 4	34	17
Parity		
<1	68	34
> = 1	132	66
Total	200	100
BMI		
<18.5	18	9
18.5–23.9	106	53
24–27.9	50	25
>27.9	26	13
Total	200	100
Maternal history of hypertension		
Yes	19	9.5
No	181	90.5
Total	200	100
Systolic blood pressure		
Normal	166	83
Elevated	34	17
Total	200	100
Diastolic blood pressure		
Normal	186	93
Elevated	14	7
Total	200	100

### Lipid profile and blood glucose level of pregnant women

Plasma sample analysis of the pregnant women showed that almost three-fourth (73.5%) and 158 (79%) had normal cholesterol and glucose levels, respectively. More than half (57%) and (55.5%) had normal level of triglyceride and low density lipoproteins, respectively while the remaining percentage was above the upper limit of the normal range (Table 3).

**Table 3:** Lipid profile and blood sugar level of pregnant women in northern Ethiopia public hospitals, 2021 [n = 200].

Variable and categories	Frequency	Percent
Cholesterol level		
0–200 mg/dl	147	73.5
>200 mg/dl	53	26.5
Total	200	100
Triglycerides		
0–150 mg/dl	114	57
>150 mg/dl	86	43
Total	200	100
Low density lipoprotein		
0–100 mg/dl	111	55.5
>100 mg/dl	89	44.5
Total	200	100
High density lipoprotein		
>99 mg/dl	10	5
55–99 mg/dl	68	34
<55 mg/dl	122	61
Total	200	100
Sugar		
74–106 mg/dl	158	79
>106 mg/dl	42	21
Total	200	100

### Lipid and glucose level of pregnant women by gestational age

Among the 53 (26.5%) pregnant women whose cholesterol level went out of the upper limit of the normal range, 30 (56.6%) were at their second trimester. Of the 86 (43%) pregnant women whose triglyceride level was out of the normal range, around two-third (65.1%) were again at their second trimester. Fifty three (26.5%) were at their second trimester period out of the 89 (44.5%) pregnant women with out of range values for low density lipoprotein. Eighteen (42.9%) were at their second trimester out of the 42 (21%) pregnant women whose glucose level was out of range (Table 4).

**Table 4:** Lipid profile and blood sugar level of pregnant women by gestational age, 2021 [n = 200].

Variables	Gestational age						Total	
	First trimester		Second trimester		Third trimester			
	n	%	n	%	n	%	n	%
Cholesterol								
0–200 mg/dl	40	20	86	43	21	10.5	147	73.5
>200 mg/dl	14	7	30	15	9	4.5	53	26.5
Triglycerides								
0–150 mg/dl	42	21	60	30	12	6	114	57
>150 mg/dl	12	6	56	28	18	9	86	43
Low density lipoprotein								
0–100 mg/dl	36	18	63	31.5	12	6	111	55.5
>100 mg/dl	18	9	53	26.5	18	9	89	44.5
Blood sugar								
74–106 mg/dl	36	18	101	52	21	10.5	158	79
>100 mg/dl	18	9	15	7.5	9	4.5	42	21

### Factors associated with lipid and glucose levels

The application of bivariate and multivariate analysis indicated variables that were associated with raised levels of lipids and glucose. In the bivariate analysis, pregnant women’s monthly income was correlated with cholesterol level; pregnant women’s age, gestational age and systolic blood pressure were correlated with total triglycerides; gestational age and systolic blood pressure were correlated with low density lipoprotein levels. In the multivariable analysis, monthly income established statistically significant association with raised level of cholesterol; age of pregnant women, gestational age and systolic blood pressure with triglycerides; and gestational age with low density lipoprotein (Table 5).

**Table 5:** Multivariable logistic regression analysis of factors associated with lipid and blood sugar levels of pregnant women in northern Ethiopia public hospitals, 2021 [n = 200].

**Cholesterol level**				
Variable	0–200 mg/dl	>200 mg/dl	COR (95%CI)	AOR (95%CI)
Mothers’ income				
< = 5,000 ETB	102	14	1	1
5,000–9999 ETB	44	8	0.93 (0.42–2.06)	0.96 (0.43–2.15)
> = 10,000 ETB	23	9	3.46 (1.53–7.85)*	3.35 (1.46–7.66)**
Diastolic blood pressure				
Normal < = 120	140	46	1	1
Elevated >120	7	7	3.04 (1.01–9.14)*	2.72 (0.87–8.50)
**Triglyceride level**				
Variables	0–150 mg/dl	>150 mg/dl	COR (95%CI)	AOR (95%CI)
Age of mothers				
18–25	64	2	1	1
26–30	82	6	3.13 (1.55–6.30)*	3.28 (1.32–8.17)**
31–38	38	8	4.06 (1.81–9.14)	3.16 (1.03–9.68)**
Gestational age				
1–16 weeks	47	7	1	1
17–28 weeks	94	22	3.27 (1.56–6.83)*	3.80 (1.68–8.57)**
29–37 weeks	23	7	5.250 (1.99–13.88)*	8.02 (2.69–23.90)*
Parity				
<1	60	8	1	1
> = 1	104	28	2.40 (1.29–4.48)*	1.62 (0.58–4.57)
Gravidity				
< = 1	41	5	1	1
2–3	95	25	2.48 (1.17–5.25)*	0.78 (0.22–2.73)
> = 4	28	6	3.19 (1.24–8.17)*	0.77 (0.17–3.56)
Systolic blood pressure				
Normal < = 120	125	41	1	1
Elevated >120	22	12	2.17 (1.02–4.58)*	3.99 (1.64–9.75)**
** Low density lipoprotein level**				
Variables	0–100 mg/dl	>100 mg/dl	COR (95%CI)	AOR (95%CI)
Gestational age				
1–16 weeks	38	16	1	
17–28 weeks	63	53	1.68 (0.86–3.30)	0.34 (0.13–0.91)**
29–37 weeks	12	18	3.00 (1.19–7.56)*	0.64 (0.28–1.50)
BMI				
<18.5	11	7	1	
18.5–23.9	65	41	0.99 (0.36–2.76)	0.68 (0.23–2.03)
24–27.9	26	24	1.34 (0.45–4.02)	1.02 (0.33–3.19)
>27.9	8	18	3.54 (1.00–12.49)*	2.42 (0.65–9.02)
Systolic blood pressure				
Normal < = 120	82	84	1	1
Elevated >120	28	6	0.19 (0.06–0.46)*	3.99 (1.64–9.75)**
**Blood sugar level**				
Variable	0–150 mg/dl	>150 mg/dl	COR (95%CI)	AOR (95%CI)
Normal < = 120	150	36	1	1
Elevated >120	8	6	3.13 (1.02–9.57)	3.13 (1.02–9.57)

The regression analysis highlighted that, pregnant women with monthly income greater than 10,000 Ethiopian Birr (ETB) had at least three times higher chance of facing raised total cholesterol level (AOR = 3.35, 95% CL: 1.46–7.66) than those whose monthly income is less than or equal to 5,000 ETB. Those with gestational age 29–37 weeks demonstrated higher odds of facing rised total triglycerides (AOR = 8.02, 95% CI: 2.69–23.90) than those with 1–16 weeks gestational age. Moreover, pregnant women with raised systolic blood pressure had greater probability of facing increased low density protein (AOR = 3.99, CI: 1.64–9.75) than those with normal systolic blood pressure.

## Discussion

Our study showed substantial rise of blood lipids particularly triglycerides and low density lipoproteins in pregnant women. Moreover, pregnant women’s age, income, gestational age and increased blood pressure are revealed to be significantly associated variables with lipids and glucose.

Our study showed that significant increase in lipids, particularly the triglycerides and low density lipoproteins, took place during pregnancy periods. Other studies conducted in USA [30, 31] and Norway [32] indicated similar results. Such increase is mostly believed to be pregnancy-induced and can impact the well-being and development of the fetus in the womb. Significant increase in concentration of triglycerides, low density lipoprotein and glucose is observed in the second trimester unlike the findings from Indian study [33] which demonstrated that further increase in the third trimester. This may suggest that monitoring the concentration of lipids and glucose during pregnancy periods, particularly the second and third trimesters, is essential for better clinical management of the fetus and the mother.

In this study, pregnant women’s monthly income was significantly associated with raised cholesterol level. This finding is consistent with a similar study conducted in Gondar, Ethiopia [34]. The study in Gondar demonstrated that pregnant women with higher monthly income had elevated total cholesterol level than non-pregnant women. This might be due to the fact that pregnant women’s demand for food almost always goes high and those who were financially in a good position tend to take much food including fatty foods like white meat which might result in elevated levels of cholesterol. Moreover, it is widely common and culturally acceptable to use ‘butter’ as part of their diet in those pregnant women with good monthly income.

This study also indicated that pregnant women’s age is statistically associated with elevated level of triglycerides, a dissimilar finding with a study conducted in China [35] where age was shown to have the reverse relation with elevated level of triglycerides. This implies that there exists a difference in socio-demographic characteristics and physical exercise practice of pregnant women. In developed nations like China, what so ever their age might be, pregnant women have different options to do physical exercise unlike pregnant women in northern Ethiopia where mothers’ tendency to do physical exercise as their age increases is poor. On the other hand, the finding that pregnant mother’s age demonstrated statistically significant association with rised levels of triglycerides was supported by a study conducted in Greece [36] in that it was proved triglycerides and total cholesterol were also influenced by pregnant women’s age.

The variable ‘gestational age’ also established statistically significant association with rised triglyceride and low density lipoprotein levels. This result is consistent with studies conducted in different nations of the globe [7–10, 36]. This suggests that the enhanced net breakdown of fat depots during late gestation is associated with hyperlipidemia, which chiefly corresponds to plasma rises in maternal triglycerides, with smaller rises in cholesterol. The rise in triglycerides and low density lipoprotein as gestational age increases is believed to be a normal occurrence and a similar situation is reflected in our study.

In our study, most of the pregnant women who demonstrated an increase in lipid levels were in their second trimester period. This finding is supported by other studies conducted elsewhere [18, 37]. This might be because of the presence of higher insulin levels and even enhanced insulin sensitivity that results in enhanced lipogenesis. In addition, increased activity of adipose tissue lipoprotein lipase during early pregnancy hydrolyzes triglycerides circulating in plasma in the form of TAG-rich lipoproteins. These all lead to the occurrence of accumulation of fat in maternal depots during the first two-thirds of gestation, i.e., the first and second trimesters of pregnancy periods [37].

The other variable that showed statistically significant association with increased triglyceride level is systolic blood pressure. Pregnant women with systolic blood pressure greater than 120 mmHg were with higher odds of having elevated triglycerides and low density lipoprotein levels than those with normal systolic blood pressure. This outcome of the present study is consistent with a study conducted in northwestern Ethiopia [34] which revealed that an increase in systolic blood pressure is statistically associated with an increase in triglycerides and low density lipoprotein levels. In pregnant women with elevated levels of lipids, it implies that the blood vessels could be constricted resulting in raised blood pressure.

## Conclusion and recommendation

High increase, unlike the other lipids, in triglycerides and low density lipoprotein is observed. Gestational age (third trimester) is a strong predictor for an increase in plasm lipids. Provision of life style related health education and dietary intake to pregnant mothers is imperative. Moreover, healthcare providers should give due attention to lipid and glucose concentration determination during their appointment periods in the antenatal care clinics.

## Study limitation

We believe that the war in Tigrai affected our study. We could have collected more samples from study participants.

## Supporting information

S1 Questionnaire Standard operating procedure for Specimen collection, handling and transport for blood lipids and glucose analysis. (DOCX)

S1 Dataset Lipids and glucose study_SPSS dataset. (SAV)
