# Supplementary material for: A facility-based study of lipids, glucose levels and their correlates among pregnant women in public hospitals of northern Ethiopia
Source: PLoS One. 2023 Jun 6;18(6):e0279595. doi: 10.1371/journal.pone.0279595 (PMC10243621; doi:10.1371/journal.pone.0279595)
Supplement: S1 Questionnaire — (DOCX) [file pone.0279595.s001.docx]

**Questionnaire**

**Part I: Demographic and socioeconomic profile**

Mother’s residence:_______________________

Zone___________________________________

District/Woreda__________________________

Tabia/Kebelle____________________________

Phone no:_______________________________

| 1 | Age of mother at 1^st^ visit | ___________________years. | |
| --- | --- | --- | --- |
| 2 | Gestational age at 1^st^ visit | ___________________weeks | |
| 3 | Occupation | - Government employee - House wife - Merchants - Farmers - College student. - Other (specify)__________________ | |
| 4 | Maternal education | 1-6 grades  7-12 grades  >12 grades | |
| 5 | Monthly family income | - <=5,000 ETB - 5,000-9999 ETB   >=10,000 ETB | |
| 6 | Do you practice physical activity? | 1. Yes  2. No | |
| 7 | If your answer to question number #8 is yes, could you list the activities | 1. _______________________________ 2. _______________________________ 3. _______________________________ 4. ________________________________ | |
| 8 | Do you take beer? | 1.Yes  2. No | |
| 9 | If your answer to question number 10 is yes, how many bottles per month? | ________________________ | |
| 10 | Do you take ‘SIWA/TEJ’? | 1.Yes  2. No | |
| 11 | If your answer to question number 14 is yes, how many Liters per month | _________________________ | |
| 12 | Do you smoke cigarette | 1.Yes  2. No | |
| 13 | If your answer to question number 16 is yes, how many packets per month? | ______________________________ | |
| **Part II: Clinical data** | | | |
|  | Gestational age | - First trimester (1-16 weeks) - Second trimester (17-28 weeks) - Third trimester (29-41 weeks) | |
| 14 | Gestational weight | __________________________Kg | |
| 15 | Maternal height | _________________ centimeter | |
| 16 | Body Mass Index (BMI) | ________________________________ | |
| 17 | Do you have history of hypertension? | 1.Yes  2.No | |
| 18 | Systolic blood pressure at enrollment? |  | |
| 19 | Diastolic blood pressure at enrollment? |  | |
| **Part III: Lipid profile and Blood glucose level** | | |  |
| 20 | Cholesterol |  | |
| 21 | Triglyceride |  | |
| 22 | Low density lipoproteins |  | |
| 23 | High density lipoproteins |  | |
| 24 | Random blood glucose |  | |
| 25 | LDL/HDL |  | |
